# Correlations between dyslipidemia and retinal parameters measured with Angio-OCT in type II diabetics without diabetic retinopathy

**DOI:** 10.22336/rjo.2024.50

**Published:** 2024

**Authors:** Geanina Totolici, Carmen Tiutiuca, Sanda Jurja, Dana Tutunaru

**Affiliations:** 1 “Dunărea de Jos” University of Galaţi, Faculty of Medicine and Pharmacy, Galaţi, Romania,; 2 “Sf. Apostol Andrei” County Clinical Emergency Hospital, Galați, Romania,; 3 “Ovidius” University of Constanţa, Faculty of Medicine and Pharmacy, Constanţa, Romania,; 4 County Clinical Emergency Hospital, Constanţa, Romania

**Keywords:** diabetic retinopathy, dyslipidemia, angio-OCT, total cholesterol, DM = diabetes mellitus, DR = diabetic retinopathy, LDL-c = LDL cholesterol, HDL-c = HDL cholesterol, CRT = central retinal thickness, DVF C = foveal vascular density, TG = triglycerides, ETDRS = Early Treatment Diabetic Retinopathy Study, CV = cardio-vascular

## Abstract

**Aim:**

To analyze the relationship between lipoproteins such as total cholesterol, LDL-c, TG, and retinal parameters in patients with DM type II without signs of DR.

**Method:**

A case-control study, consisting of 2 groups. A group of 64 patients with type II diabetes without signs of DR and a control group of 24 healthy subjects. Patients with DM type I, those who showed signs of DR, and those who had associated other eye diseases were excluded.

**Results:**

The patients of the two studied groups had a similar average age: 65 years in the DM type II group and 64 years in the control group. In the group with DM, the average CRT was 241.31 µm, a significantly lower value compared to the control group, 252.51. The average value of DVFC was 19.19%, in patients with DM and 24.29% in the control group. An indirect correlation with moderate intensity was established between total cholesterol and CRT, (rs=–0.442, p≤0.001), thus it tended to decrease as total cholesterol increased. With increasing total cholesterol level, DVFC had a mild tendency to decrease (rs=–0.381, p≤0.001). An indirect correlation, but weak in intensity, existed between the LDL/HDL ratio and the DVFC S value (rs=–0.240, p=0.001).

**Discussions:**

Central retinal thickness and central vascular density of the superficial capillary plexus were significantly lower in patients with type II diabetes, compared to control subjects. Total cholesterol had higher values in the DM group and an indirect correlation was established with CRT and DVFC, these having a moderate tendency to decrease as the total cholesterol values increased. An indirect and moderate relationship in intensity was also present between LDL and retinal parameters studied. These results were similar to those of other studies conducted, such as that of Chen et al. or Bernaous et al., who showed an association between various lipid classes and the frequency of DR. However, other studies, such as Ausdiab, found that this association did not hold.

**Conclusions:**

Type II diabetes patients tend to have elevated serum lipid levels compared to normal subjects, but the impact of dyslipidemia on the onset and progression of DR is incompletely elucidated.

## Introduction

Diabetes mellitus (DM) is a chronic disease, whose global prevalence is continuously increasing and that statistical data predict will reach approximately 12.2% by the year 2045, i.e. 783 million patients [1,2].

The high mortality and morbidity associated with diabetes make it a public health problem, as it threatens patients’ quality of life and the global economy through high associated costs [1,3,4].

Diabetic retinopathy (DR) is not only a microvascular but also a neurovascular complication, which affects over 80% of patients with diabetes, whose evolution exceeds 20 years, being the main cause of blindness among the active population [5,6].

Although hyperglycemia, age of diabetes, microalbuminuria, and blood pressure levels are well-known risk factors for the development of DR, there is significant individual variation in its occurrence and progression. Therefore, it is necessary to identify additional risk factors to prevent and reduce the prevalence of DR [5,7,8].

The link between lipid metabolism and the prognosis of cardiovascular diseases has been intensively studied, clearly establishing the fact that dyslipidemia represents a risk factor. Instead, the impact of lipid metabolism on DR has been less studied and the results are contradictory [1,9-13].

### Purpose

The study aimed to quantify the impact of dyslipidemia on retinal parameters in patients with type II diabetes, who do not show signs of diabetic retinopathy on fundus examination.

### Objectives

- Analysis of the relationship between total cholesterol, LDL-cholesterol (LDL-c), triglycerides (TG) and central retinal thickness (CRT);
- Correlation between total cholesterol, LDL-cholesterol, TG, and superficial central vascular density (DVC S);
- The relationship between LDL-c/HDL-c ratio and retinal parameters: CRT and DVC S.

### Method

This was a retrospective case-control type study, carried out in the Ophthalmology Department of “Sf. Apostol Andrei” County Clinical Emergency Hospital, in Galaţi, during 2021-2022.

Inclusion criteria included:

- Age over 18 years;
- Patients diagnosed with DM type II.

From the total number of patients who met the inclusion conditions, we selected a lot of 64 patients following the application of the exclusion criteria.

The exclusion criteria were:

- Type I DM diagnosis;
- Diabetic retinopathy at any stage;
- The presence of associated cardiovascular diseases;
- Age under 18 years;
- Mature cataract, corneal leukoma, vitreous hemorrhage, hemophthalmos, and other eye conditions that did not allow visualization of the retina; -
- Other associated retinal conditions.

A control group composed of clinically and paraclinically healthy subjects was also selected.

All subjects enrolled in the study were examined from an ophthalmological point of view, noting the following data: visual acuity, intraocular pressure, and refraction. Angio-OCT was performed to determine central retinal thickness and central superficial vascular density. The angio-OCT examination was performed with Topcon Maestro II and the macular area scanned was 6 mm x 6 mm, based on ETDRS charts.

To determine the serum values of total cholesterol, LDL-c, HDL-c, and triglycerides, we used Konelab 60I Prime.

The general data of the patients were collected: age, sex, environment, and body mass index.

The study was conducted with the approval of the ethics committee. An informed consent was obtained from the patients.

The data were analyzed using the IBM SPSS Statistics program version 21. The statistical tests used in the analysis were: the Mann-Whitney U test, Chi-Square test, Phi Coefficient, and Spearman’s correlation. The normality of distributions was determined using the Shapiro-Wilk test and graphical analysis. Values of p<0.05 were considered statistically significant. The data were guaranteed with a probability of 95% and a maximum permissible error limit of ±5%.

## Results

The general characteristics of the subjects included in the study are presented in **Table 1**.

**Table 1:** General characteristics among the study subjects

Parameters	Lot DM (n=64)	Control (n=24)
Age (years, average, intervals)	65.12 (51-78)	64.10 (48-77)
Men	33	10
Women	31	14
BMI (average, intervals)	25.79 (22-32)	23.28 (22-24)
Total cholesterol (mg/dl, average, intervals)	237.70 (130-318)	200.49 (173-218)
HDL-c (mg/dl, average, intervals)	60.46 (46-89)	80.14 (75-93)
LDL-c (mg/dl, average, intervals)	144.74 (85-240)	92.81 (85-100)
LDL-c/HDL-c ratio	2.40 (1.5-3.8)	1.08 (0.9-1.1)
TG (mg/dl, average, intervals)	158.59 (105-270)	127.46 (111-140)
CRT (μm, average, intervals)	241.31 (216-330)	252.21 (244-262)
DVFC (%, average, intervals)	19.19 (10.24-23.19)	24.29 (23.10-27.52)

The study included 88 patients, 64 with DM (study group), and 24 normal patients (control group). The average age in the group of patients with DM was 65.12 years and in the control group 64.10 years.

### BMI

In the control group, all patients had normal weight (100%), while in the group with DM patients were most frequently overweight or class 1 obese (61%), and only 39% had a normal weight.

**Fig. 1:**
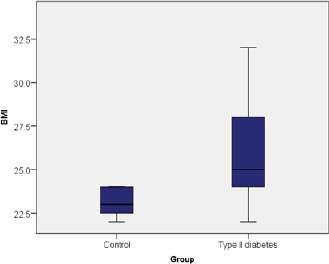
Comparison of BMI values in the two groups

The chi-square test indicated that the BMI of diabetic patients differed significantly from that of patients without pathologies, with diabetics having a strong tendency to present a higher BMI (χ2=68.507, p≤0.001) (**Fig. 1**).

### Central retinal thickness (CRT)

Central retinal thickness, or foveal thickness represents the average thickness in the central area after applying the Early Treatment Diabetic Retinopathy Study (ETDRS) grid [14].

**Fig. 2:**
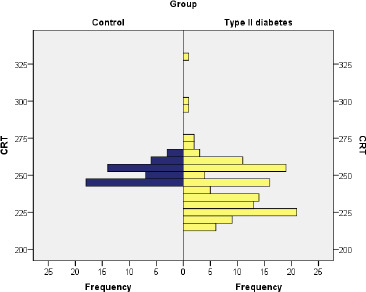
Compaison of CRT among control group and type II diabetes

The patients in the DM group had an average value of the CRT parameter of 241.31 µm, with values between 216 µm and 330 µm, with men showing significantly lower values compared to women (average value 236.33 µm vs. 245.72 µm) (U=1372, Z =–3.218, p=0.001). Patients in the control group had an average value of 252.21 µm, presenting values between 244 µm and 262 µm (**Fig. 2**).

Mann-Whitney U test was applied to determine if there were differences in CRT between the DM group and the control group, indicating that the presence of diabetes correlated with a significant decrease in CRT in the studied patients (U=1.651, Z =–4.725, p≤0.001).

### Central vascular density (DVF C)

Central vascular density is defined as the vascular density of the superficial capillary plexus in the central area after applying the ETDRS grid.

The patients in the DM group had an average value of the DVF C parameter of 19.19%, being between 10.24% and 23.19%, and the patients in the control group had an average value of 24.29%, the DVFC S being between 23.10% and 27.52%. The values were similar between the sexes.

**Fig. 3:**
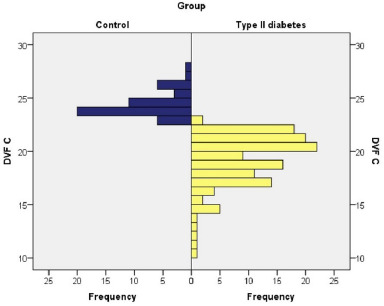
Comparison of DVF C among control group and type II diabetes

Mann-Whitney U indicated that diabetes correlated with a significant decrease in DVF C S in the studied patients (U=5,000, Z=–10.189, p≤0.001) **(Fig. 3**).

### Total cholesterol

Diabetic patients were characterized by increased levels of total cholesterol, with an average value of 237.70 mg/dl compared to 200.49 mg/dl in the control group. Among diabetic patients, 50% had elevated total cholesterol, 30.50% borderline, and only 19.50% within normal parameters.

**Fig. 4:**
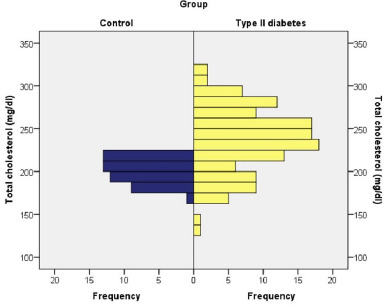
Total cholesterol values in control group and type II diabetes

The Chi-square test revealed a statistically significant association between the presence of diabetes and the level of total cholesterol, which was direct and with moderate intensity (χ2=51.042, coeff. Phi=0.438, p≤0.001). So, diabetic patients tended to have significantly higher total cholesterol levels than patients without pathologies (**Fig. 4**).

**Fig. 5:**
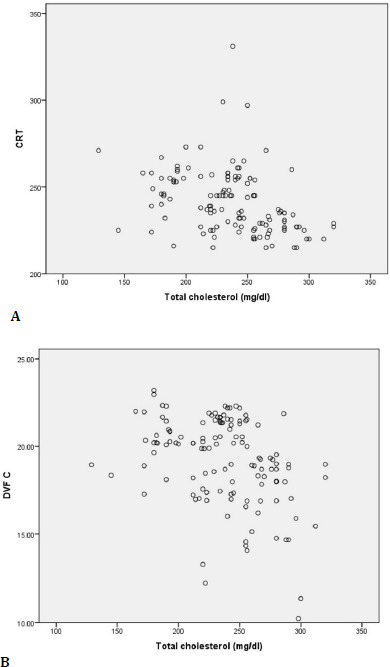
Correlations between total cholesterol and CRT **(A**) , and DVF C (**B**)

A Spearman correlation coefficient was calculated to assess the relationship between total cholesterol level and CRT value in DM patients. The graphical representation indicated a monotonic relationship. There was an indirect correlation with moderate intensity between total cholesterol and CRT, (rs=– 0.442, p≤0.001), so the diabetic patients’ CRT tended to decrease moderately as total cholesterol increased **(Fig. 5 A**) .

Moreover, an indirect and moderate correlation in intensity was also established between the total cholesterol and the DVFC value, so that with the increase in the total cholesterol level, the DVFC had a moderate tendency to decrease (rs=–0.38, p≤0.001) **(Fig. 5 B**) .

These trends were not observed in patients in the control group.

### LDL-c

**Fig. 6:**
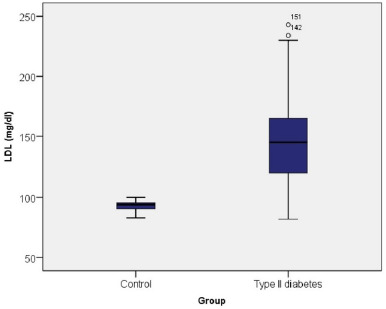
LDL-c values among control group and type II diabetes

Diabetic patients presented an average LDL-c level of 144.74 mg/dl, significantly higher than in the control group (92.81 mg/dl). In 35.90% of cases, the LDL-c level indicated an increased cardiovascular (CV) risk, in 51.60% a moderate risk, respectively in only 12.50% of the cases LDL-c was within limits (**Fig. 6**).

The Chi-square test indicated that there was a statistically significant association between the presence of DM and the LDL-c level, diabetics being prone to have a significantly higher CV risk compared to patients without pathologies, who tend to have optimal LDL values (χ=115.503, p≤0.001).

**Fig. 7:**
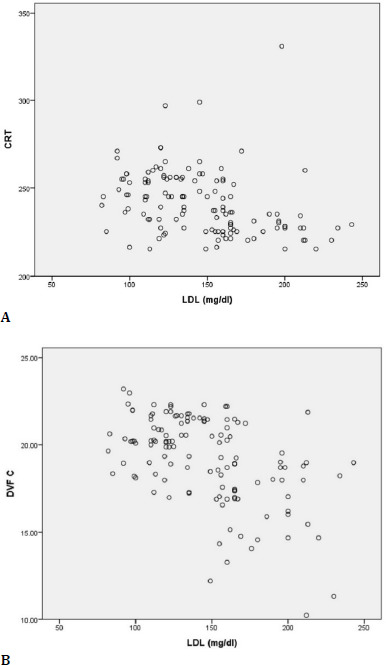
Correlations between LDL-c and CRT (**A**) and DVFC (**B**)

Also, the calculation of a Spearman coefficient to evaluate the relationship between LDL-c and CRT value in DM patients indicated that there was an indirect and moderate correlation between these parameters, (rs=–0.427, p≤0.001), so as diabetic patients had higher LDL-c levels, and CRT had a mild tendency to decrease (**Fig. 7 A**).

The same indirect and moderate correlation in intensity was established between the LDL level and the DVFC value. With the increase in the LDL-c level, the DVFC had a mild tendency to decrease (rs=–0.477, p≤0.001) (**Fig. 7 B**).

### LDL-c/HDL-c ratio

**Fig. 8:**
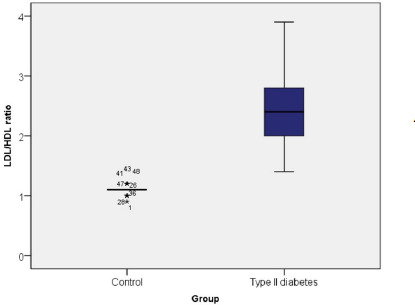
LDL-c/HDL-c ratio in the control group and type II diabetes

LDL cholesterol provides cholesterol transport to the tissues, mainly at the arterial level, which leads to atherosclerosis and coronary heart disease in patients with elevated serum levels of this lipoprotein [15,16]. Thus, LDL-c determination is useful for estimating cardiovascular risk and establishing therapeutic behavior [17,18]. Under physiological conditions, the LDL-c/HDL-c ratio is 2.9 in women and 3.3 in men. The risk of developing cardiovascular disease increases significantly when the value of this ratio exceeds 3.5 in women and 3.8 in men [19] (**Fig. 8**).

A considerable increase in the LDL-c/HDL-c ratio was noted in patients with DM compared to healthy ones (2.40 vs. 1.08), so diabetics had increased CV risk in 0.80% of cases, moderate CV risk in 11.70% of cases, and an LDL-c/HDL-c ratio within normal limits in 87.50% of cases. A Mann-Whitney U test was applied to determine whether there were differences in the LDL-c/HDL-c ratio between the DM group and the control group, indicating that the presence of diabetes correlated with a significant increase in the LDL-c/HDL-c ratio (U=6.144, Z=–10.233, p≤0.001).

**Fig. 9:**
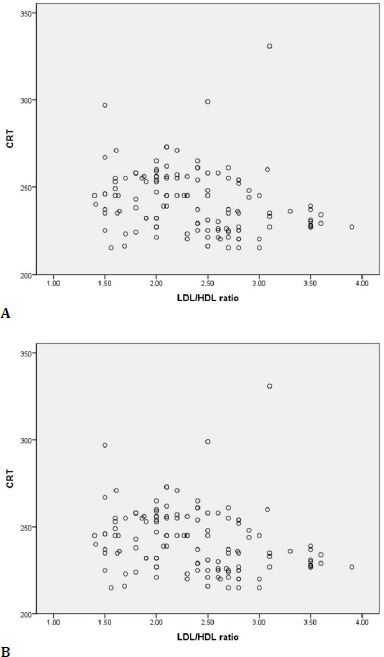
Correlations between LDL-c/HDL-c ratio and CRT **(A**) and DVF C (**B**)

A Spearman correlation coefficient was calculated to assess the relationship between LDL-c/HDL-c ratio and CRT in DM patients. The graphical representation indicated a monotonic relationship. There was an indirect correlation with moderate intensity between the LDL-c/HDL-c ratio and CRT, (rs=–0.327, p≤0.001), so in the diabetic patient, CRT tended to decrease moderately as the LDL-c/HDL-c ratio increased (**Fig. 9 A**).

An indirect correlation, but weak in intensity, existed between the LDL-c/HDL-c ratio and the DVFC S value so that with the increase in the value of the ratio, the DVFC S had a slight tendency to decrease (rs=–0.240, p=0.001) (**Fig. 9 B**).

### Triglycerides

An increase in the level of TG was observed in the case of diabetic patients compared to patients in the control group (158.59 mg/dl vs. 127.46 mg/dl) so that 8.60% of patients with DM had an increased level, 41.40% borderline values, respectively 50% normal values.

**Fig. 10:**
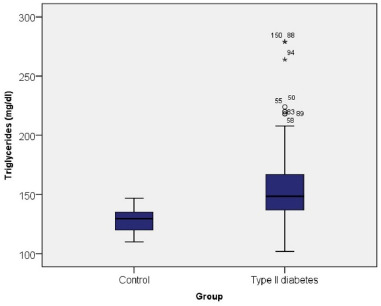
TG values among the control group and type II diabetes

The Chi-square test indicated a direct association between the presence of diabetes and the level of TG, with diabetic patients having a moderate tendency to present significantly higher values (χ2=39.184, coeff. Phi=0.392, p≤0.001) (**Fig. 10**).

**Fig. 11:**
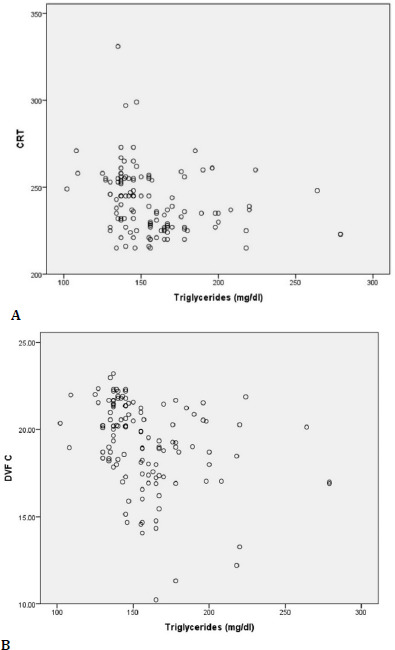
Correlations between TG and CRT (**A**), and DVF C (**B**)

The Spearman coefficient to evaluate the relationship between triglycerides and CRT value in DM patients indicated that there was an indirect and moderate correlation between these parameters, (rs=–0.338, p≤0.001), so as diabetic patients had a higher triglyceride level and CRT had a mild tendency to decrease (**Fig. 11 A**).

The same coefficient indicated that in the case of diabetic patients there was an indirect and moderate correlation in intensity between the level of triglycerides and the value of DVFC S so that together with triglycerides, DVFC S had a mild tendency to decrease (rs=–0.430, p≤0.001) (**Fig. 11 B**).

## Discussions

Retinal vascularization is the only deep microvascular system, which can be directly observed by performing the fundus examination [1,20]. Structural and functional changes in the retina may represent a predictive factor, in terms of the occurrence, of micro and macrovascular complications of DM, thus leading to the early diagnosis of DR [1,21].

The present study tries to clarify the impact of dyslipidemia on DR by analyzing the relationship between total cholesterol, LDL-c, LDL-c/HDL-c ratio, TG, and retinal parameters such as CRT and DVFC.

The patients’ ages were similar in the DM and the control group. Instead, most patients in the DM group had an increased BDI compared to the control group, composed only of normal-weight subjects.

The group with DM had a significantly lower CRT after the statistical test application. These results were similar to those of other studies. Jing et al. reported a significant retina thinning in the foveal, perifoveal, and parafoveal temporal area, in patients with DM, but who did not show signs of DR compared to the control group [22,23].

Also, DVFC was significantly reduced in patients with DM compared to the control group. The results of the study published in 2022, by Jingjie Liu et al., reported similar results [24].

Analyzing the total cholesterol values, it was found that 50% of patients with DM had above the normal limit, 30.5% at the limit, and only 19.5% normal values. An indirect correlation of moderate intensity was established between total cholesterol and retinal parameters. Thus, both CRT and DVFC had a mild tendency to decrease as total cholesterol values increased.

LDL-c values indicated that more than 80% of DM patients had moderate-increased CV risk. These results are similar to those in the literature that mention LDL-c and DM as CV risk factors.

Moreover, LDL-c was statistically correlated with CRT and DVFC, showing a slight tendency for retinal parameters to decrease when LDL-c values increased.

An indirect, but weak, relationship was established between the LDL-c/HDL-c ratio and retinal parameters. There was also an indirect, but moderate, relationship between TG levels and studied retinal parameters.

The results presented above are similar to those of studies in the literature that reported an association between different classes of lipids and the appearance of retinal changes specific to DR [25,26].

For example, by multivariate Cox regression, Chen et al. reported TG as a risk factor for DR. Also, increased total cholesterol values were associated with the incidence of DR and EMD and increased TG levels were associated with progression to proliferative DR [27-29].

At the same time, Beanrous et al. showed that patients with elevated LDL-c levels had an increased risk of developing clinically significant macular edema [27,30,31].

Considering studies such as Ausdiab, with opposite results, to those previously described, the relationship between serum lipids and DR remains incompletely elucidated [3-12].

### Study strengths and limitations

The strong point was the inclusion in the study of only patients with DM, without other associated cardiovascular diseases, precisely to reduce the direct influence they could have on the morphological changes of the retina.

One of the weak points of the study was the small number of participants. Moreover, no data regarding lipid-lowering treatment were collected, so we could not assess its influence on the results.

## Conclusions

Dyslipidemia is a condition frequently associated with diabetes. In the case of patients with diabetes, retinal parameters are modified, in the sense of reducing both the central thickness of the retina and the central vascular density of the superficial capillary plexus, even in the absence of signs of diabetic retinopathy.

Dyslipidemia was also associated with morphological changes in the retinal layers, but its impact was weak in intensity.

More prospective studies are needed for a more detailed assessment of the role that dyslipidemia has on the onset and progression of DR.
